# Movement patterns and habitat use for the sympatric species: *Gambelia wislizenii* and *Aspidoscelis tigris*

**DOI:** 10.1002/ece3.10422

**Published:** 2023-08-10

**Authors:** Elizabeth McAlpine‐Bellis, Kaera L. Utsumi, Kelly M. Diamond, Janine Klein, Sophia Gilbert‐Smith, Grace E. Garrison, Maria A. Eifler, Douglas A. Eifler

**Affiliations:** ^1^ Erell Institute Lawrence Kansas USA; ^2^ Biodiversity Institute University of Kansas Lawrence Kansas USA; ^3^ Department of Biology Rhodes College Memphis Tennessee USA; ^4^ Department of Anthropology University of California Santa Barbara California USA

**Keywords:** Great Basin Desert, path segmentation, resource partitioning, search behavior, sensory ecology

## Abstract

Movement is an important characteristic of an animal's ecology, reflecting the perception of and response to environmental conditions. To effectively search for food, movement patterns likely depend on habitat characteristics and the sensory systems used to find prey. We examined movements associated with foraging for two sympatric species of lizards inhabiting the Great Basin Desert of southeastern Oregon. The two species have largely overlapping diets but find prey via different sensory cues, which link to their differing foraging strategies—the long‐nosed leopard lizard, *Gambelia wislizenii*, is a visually‐oriented predator, while the western whiptail, *Aspidoscelis tigris*, relies more heavily on chemosensory cues to find prey. Using detailed focal observations, we characterized the habitat use and movement paths of each species. We placed markers at the location of focal animals every minute for the duration of each 30‐min observation. Afterward, we recorded whether each location was in the open or in vegetation, as well as the movement metrics of step length, path length, net displacement, straightness index, and turn angle, and then made statistical comparisons between the two species. The visual forager spent more time in open areas, moved less frequently over shorter distances, and differed in patterns of plant use compared to the chemosensory forager. Path characteristics of step length and turn angle differed between species. The visual predator moved in a way that was consistent with the notion that they require a clear visual path to stalk prey whereas the movement of the chemosensory predator increased their chances of detecting prey by venturing further into vegetation. Sympatric species can partition limited resources through differences in search behavior and habitat use.

## Graphical abstract

Through detailed behavioral observations, we characterize the movement paths and habitat use of two sympatric lizard species to test the hypothesis that visual versus chemosensory prey detection is associated with different space use and movement patterns. In addition to differences in step length and turn angles, the visual forager spent more time in the open, moved less frequently over shorter distances, and differed in plant use compared to the chemosensory forager. Sympatric species with similar diets can partition resources through differences in space use and movement.

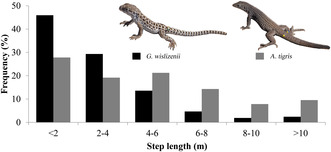

## 1 INTRODUCTION

Animal movement involves dynamic interactions that balance the organism's capabilities and ecological preferences with local environmental conditions. Their ability to move can depend not only on their locomotor structures, but also on their ability to detect and respond to current conditions (Higham, 2007). On large spatiotemporal scales, movement patterns influence population distributions as well as interactions among species and their environment (Krauel et al., 2018; Nathan et al., 2008; Smouse et al., 2010). At the individual level, when and where organisms move can directly influence survival and ultimately fitness (Cooper & Frederick, 2007; Wearmouth et al., 2014). Fine‐scale locality data coupled with movement path analyses can identify the factors influencing movement as well as how individual animals perceive and respond to their environment (Edelhoff et al., 2016; Kays et al., 2015; Nathan et al., 2008; Schick et al., 2008).

Over the past several decades, studies on animal movement have provided insights into the evolutionary biology, ecology, and physiology of many different taxa (Huey & Pianka, 1981; Miles et al., 2007; O'Brien et al., 1990; Perry, 2007; Sunquist & Montgomery, 1973). Similar movement patterns can be employed by multiple taxa, facing similar ecological needs (Abrahms et al., 2017; O'Brien et al., 1990; Symes et al., 2013). Examining spatiotemporal movement has led to significant insights into social behavior (Leu et al., 2016). Movement indices reveal intraspecific variation (Childers & Eifler, 2015; Eifler et al., 2007; Garrison et al., 2017; Huey & Pianka, 1981; Perry, 2007) or behavioral flexibility (Durtsche, 1992; Eifler et al., 2008; Eifler & Eifler, 1999; Greeff & Whiting, 2000) in foraging. Interspecific variation in foraging movement can be associated with differences in diet, space use, or habitat selection among sympatric species (Kozlowski et al., 2006; Parra, 2006; Waite et al., 2012). Additionally, variations in habitat structure and resource use can lead to changes in movement patterns (Attum & Eason, 2006; Colombo et al., 2016; Donihue, 2016; Morice et al., 2013; Wasiolka, Blaum, et al., 2009; Wasiolka, Jeltsch, et al., 2009). Using a fine‐scale spatial approach to studying movement patterns, we aim to elucidate how two sympatric lizard species can partition overlapping food resources.

Comparing the movement and behavior of different species can be difficult, yet an understanding of how each species coexists within the same habitat can reveal patterns of coevolution between species, as well as influence conservation decisions (Cooper, 1994; Cooper et al., 2001; McLaughlin, 1989; Pietruszka, 1986). In all ecosystems, competition and natural trophic stratification mean that coexisting species must find and occupy a different ecological niche to survive. In the Great Basin Desert in southeastern Oregon, two sympatric species of lizard, the long‐nosed leopard lizard (*Gambelia wislizenii*) and the western whiptail (*Aspidoscelis tigris*), present an interesting contrast. While phylogenetically in distant clades (Iguania vs. Scleroglossa) (Tonini et al., 2016), *G. wislizenii* and *A. tigris* provide an opportunity to compare how two cohabitating species behaviorally partition largely overlapping resources. A previous comparison of these species in the same location showed differences in behavior between the lizards, with *G. wislizenii* spending less time moving than *A. tigris* (McElroy et al., 2011). We build on the previous comparison of overall movement levels by analyzing movement patterns and resource acquisition from a habitat‐use perspective.

In the southwestern United States, the two species largely overlap in desert habitats with finite resources where the harsh environment requires animals to take advantage of any available food or shelter (Grismer, 2002; Hammerson, 1999). Their shared diet and habitat allowed us to make an interspecies comparison of movement ecology. The two species share an overlapping diet consisting of grasshoppers, beetles, spiders, antlions, and caterpillars differing slightly in their food choices, as *G. wislizenii*, with their larger body size, can prey on small lizards while *A. tigris* are proficient at digging for termites and buried larvae (Cooper et al., 2001; Grismer, 2002; Hammerson, 1999; McElroy et al., 2011; Tonini et al., 2016). They also share predators (snakes, birds, and desert mammals) (Grismer, 2002), and exist in the same spatial and temporal niche.

Despite sharing habitats and consuming similar prey, the species vary in their foraging strategies and rely differently on sensory modalities for prey detection (Anderson, 1993; Montanucci, 1967; Parker & Pianka, 1976; Pianka, 1970). Leopard lizards primarily use visual cues for finding food, pursuing prey that moves within their visual detection range (≤10 m away) (Anderson, 2007; Cooper, 1995; Garrison et al., 2017; Tollestrup, 1983). The movement rate for leopard lizards can vary with environmental conditions and among individuals (Anderson, 2007; Garrison et al., 2017). In contrast, whiptail movement is wide‐ranging and can vary with habitat structure (Anderson & Karasov, 1988; Utsumi et al., 2020). Further, whiptails primarily locate prey (even below the surface) through chemical sampling, only sometimes using vision to detect food items (Anderson, 1993; Cooper & Whiting, 2000; Utsumi et al., 2020).

Finding associations between prey detection strategies and differences in movement patterns can help identify the importance of sensory systems in shaping both foraging strategies and interactions between potential competitors. In terms of the movement characteristics of foraging modes, chemosensory foragers tend to move more frequently and spend a higher proportion of time moving compared to visual hunters (Baeckens et al., 2017; Eifler et al., 2020). However, the foraging mode does not necessarily indicate the size of the area searched or the path traveled. Our overarching hypothesis is that differences in prey detection are associated with the species' space use and movement patterns. Our goal was to determine if there were consistent differences in habitat use and movement patterns between animals that generally use different prey detection cues: visual long‐nosed leopard lizards and chemosensory western whiptails. We predicted that a more visually‐oriented predator would move along straighter paths, travel less, and make more use of open spaces than a chemosensory forager.

## 2 METHODS

Our study was conducted in the desert scrub habitat of the Alvord Basin, located in the Great Basin Desert, southeastern Oregon (42°18′ N, 118°37′ W; datum = WGS 84; elevation 1295 m) from 20 June to 14 July 2017. The study site was a 16‐ha gridded plot characterized by open desert sand and hardpan interspersed with patches of shrubs, mainly sage (*Artemisia tridentata*) and greasewood (*Sarcobatus vermiculatus*). We conducted observations on the long‐nosed leopard lizard, *G. wislizenii* (visual predator; *N* = 61), and the western whiptail, *A. tigris* (chemosensory predator; *N* = 51), during their morning activity period (08:00–10:30 h). Each observation was conducted by a pair of observers at a distance that allowed for clear observation without disturbing foraging behavior (ca. 2–5 m) (Anderson, 1993; Eifler et al., 2020; Utsumi et al., 2020). Prior to data collection, we conducted focal observations of lizards off‐site to assess the distance needed to minimize disturbance. Observers kept a minimum distance of 2 m away from focal lizards unless lizards actively moved closer to the observers, in which case observers did not move until lizards were again at least 2 m away. Observations lasted 30 min with one observer tracking lizard movements and the other observer placing markers at the lizard's location at each 1‐min interval. To minimize disturbance, markers were placed after the animal left the immediate area (>2 m) where a marker was to be placed. In instances where the lizard moved short distances between minutes, observers took notes in field books to ensure markers were placed in the correct locations. For each marker, we recorded whether the location was in vegetation or the open. When the location was in vegetation, we recorded the plant species. Unmarked lizards were captured using a lasso attached to an extendable pole, measured (body mass via Pesola scales [g] and clear rulers for snout–vent length [SVL; mm]), and marked with a unique paint code before being released at the capture site. Lizards were sexed by probing for the presence or absence of hemipene pockets. We recorded movement data only once for each individual animal.

### 2.1 Habitat and movement analysis

A full observation generated 31 location‐time points for each animal, consisting of x‐y coordinates and associated vegetation measurements. From the sequence of locations, we calculated the path characteristics of *step length*, *path length*, *net displacement*, *straightness index*, and *turn angle* (Table 1). We considered movement variables for each 1‐min interval. When the animal did not move during an interval, that period was not used for analysis but used to determine the proportion of periods of no movement. Several observations did not last the full 30 min, in which case we did not calculate path length and net displacement. In addition, we calculated a *visibility index*, measured as the proportion of locations from which the next location was visible to the lizard. We deemed a location to be visible to the lizard from the previous location if the line between the two locations was free of vegetation at the ground level (i.e., from the lizard's line of sight). To measure visibility, a researcher sighted along the horizon just above the substrate over one location marker in the direction of the next location marker, allowing us to assess what the animal could see from each vantage point and assess whether the direction taken had a clear or obstructed view. We recorded each sequential step in the lizard's path as either clear or obstructed, then determined the proportion of locations that were clear for each observation (i.e., visibility index). We used air temperature and wind speed measures obtained from a weather station adjacent to our site to estimate local conditions at each 1‐min interval of an observation.

**TABLE 1:** Definitions of the movement characteristics we measured.

Measurement	Definition
Step length	The straight‐line distance between consecutive 1‐min locations
Path length	The sum of all step lengths for an observation period.
Net displacement	The straight‐line distance between initial and final locations
Straightness index	The ratio of net displacement to path length (value from 0 to 1)
Turn angle	The change in direction between consecutive steps (value from 0° to 180°, with 0° = orientation of the focal animal at the previous step)

### 2.2 Data analyses

We used Minitab 18 (College Park, PA) for most analyses and R (Batschelet, 1981; R Development Core Team, 2017) for circular statistics, applying a significance level of *p* ≤ .05 to all tests. To examine the distributions of step length and turn angle as well as habitat use patterns, we pooled the measurements by species. We tested data for normality and applied non‐parametric Mann–Whitney tests to pairwise comparisons of data that were not normally distributed. We tested for species differences in habitat use (species of plant visited) using chi‐square analyses. To test for differences in step length and turn angle distributions we conducted a two‐sample Kolmogorov–Smirnov test. We applied circular statistics to comparisons of mean turn angles, and to test for uniformity and conformity to von Mises distributions. We examined within‐species variation in step length using a mixed‐effects model with lizard individual identifier as a random factor and sex, body size (SVL), and weather variables as fixed factors. We did not observe any behavioral change in the lizards during the duration of our observations in the field. We tested for a possible observer effect using a general linear model (GLM) to examine if animal movements varied over the course of the observation period, with time in the observation period as our independent variable.

## 3 RESULTS

### 3.1 Movement patterns

**FIGURE 1:**
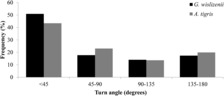
Distribution of turn angles for *Gambelia wislizenii* (visual forager; black) and *Aspidoscelis tigris* (chemosensory forager; gray), separated into bins of 45°.

**FIGURE 2:**
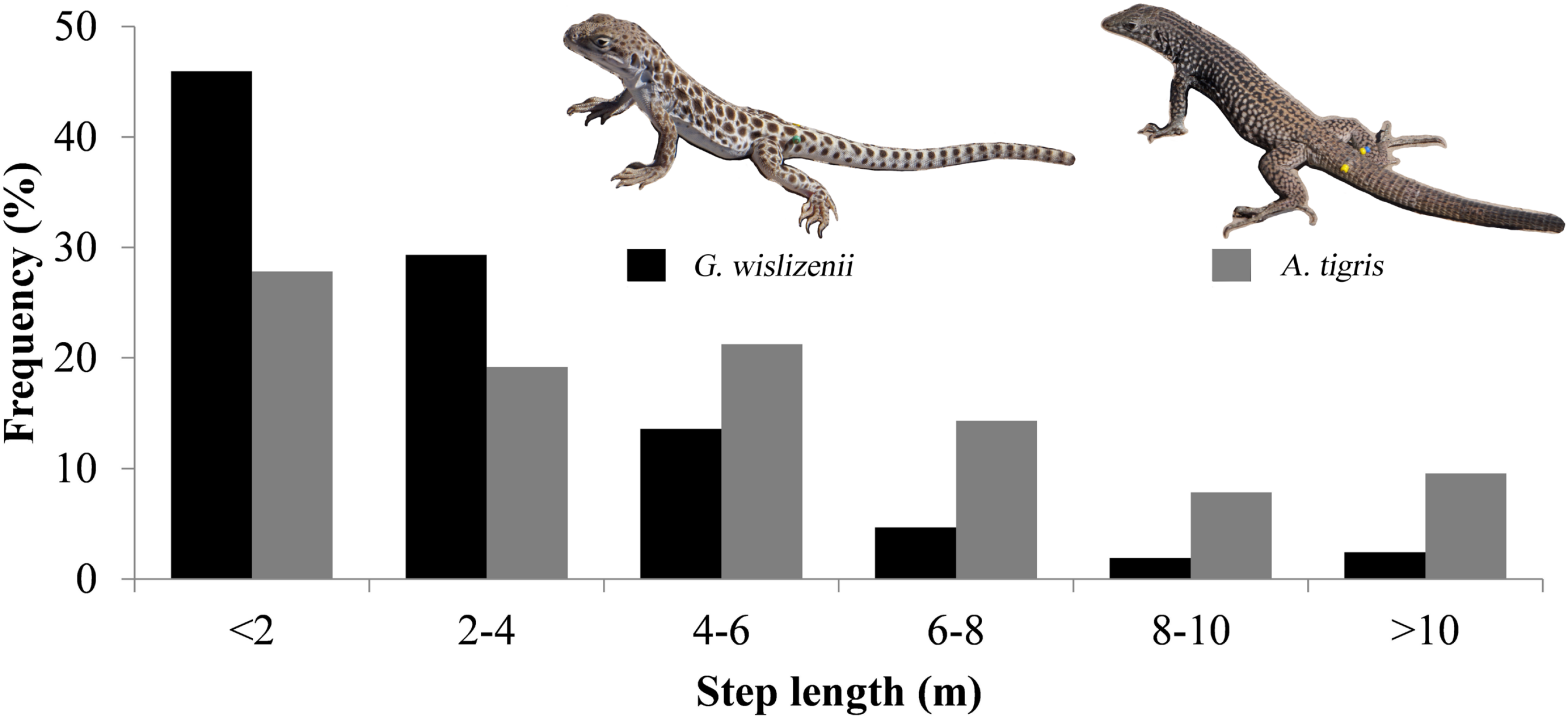
Distribution of step lengths for *Gambelia wislizenii* (visual forager; black) and *Aspidoscelis tigris* (chemosensory forager; gray).

The two species differed in most characteristics of their movement paths including turn angle, straightness index, step length, and path length (Table 2). The distribution of turn angles for both species was significantly different from a uniform circular distribution (Rayleigh's uniformity test: visual predator (long‐nosed leopard lizards, *G. wislizenii*): *z* = 69.1, df = 568, *p* < .001; chemosensory predator (western whiptails, *A. tigris*): *z* = 81.85, df = 1004, *p* < .001). The distribution of turn angles and median turn angle differed between the species (Kolmogorov–Smirnov test: *D* = 0.087, *p* = .0073; Figure 1; Table 2), with the visual predator making a greater proportion of forward‐directed movements and having smaller median turn angles. The strength of directionality (mean vector length) was 0.34 for the visual predator and 0.28 for the chemosensory predator. Leopard lizards moved less frequently than whiptails (visual predator: 44% vs. chemosensory predator: 75% of intervals had movement; *z* = 16.97, *p* < .001). During intervals of movement, the median step length was half as long for the visual predator (Table 2) and the distribution of step lengths differed between the species, with the visual predator being less likely to have longer step lengths (Kolmogorov–Smirnov test: *D* = 0.303, *p* < .0001; Figure 2).

**TABLE 2:** Summary of movement parameters (median (range)) for *Gambelia wislizenii* (visual forager) and *Aspidoscelis tigris* (chemosensory forager), compared using Mann–Whitney tests.

Measurement	*G. wislizenii*	*A. tigris*	*U*	*p*
Step length (m)	2.1 (0.02–45.3)	4.2 (0.01–42.8)	1,120,731	**<.0001**
Path length (m)	28.9 (0.0–203.1)	92.3 (2.2–251.3)	2537	**<.001**
Net displacement (m)	16.6 (0.0–116.5)	21.5 (1.3–126.3)	2005	.243
Straightness index	0.663 (0.09–1.00)	0.308 (0.02–0.92)	1095	**<.001**
Turn angle	44° (0–180)	53° (0–180)	820,076	**.010**

*Note*: Statistically signficant *p*‐values are in bold.

**FIGURE 3:**
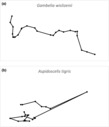
Representative paths from observed *Gambelia wislizenii* (visual forager; a) and *Aspidoscelis tigris* (chemosensory forager; b). Both images are to the same scale.

Median path length also was shorter for the visual predator than for the chemosensory predator (Table 2), indicating that the chemosensory lizard moved greater overall distances during observations. However, median net displacement was comparable for both species (Table 2), indicating similarity in search area. Path straightness varied by species, with the visual predator traveling straighter paths (Table 2; Figure 3). Turn angle and step length were not correlated for either species (visual predator: *r* = .055, *p* = .090; chemosensory predator: *r* = −.031, *p* = .474). Step length for the chemosensory predator was greater for larger animals (SVL: *F*_1,41_ = 9.77, *p* = .003) and unrelated to sex, wind speeds, or air temperatures. For the visual predator, step length was unrelated to sex, SVL, or weather variables.

The movement pattern of an individual, as indicated by step lengths and turn angles, did not vary for either species over the course of an observation, indicating that our presence did not affect lizard behavior (GLM; *G. wislizenii* step length: *F*_1,1714_ = 0.02, *p* = .875 and turn angle: *F*_1,476_ = 0.4, *p* = .529; *A. tigris* step length: *F*_1,1446_ = 0.3, *p* = .583 and turn angle: *F*_1,911_ = 0.43, *p* = .511).

### 3.2 Habitat use

**FIGURE 4:**
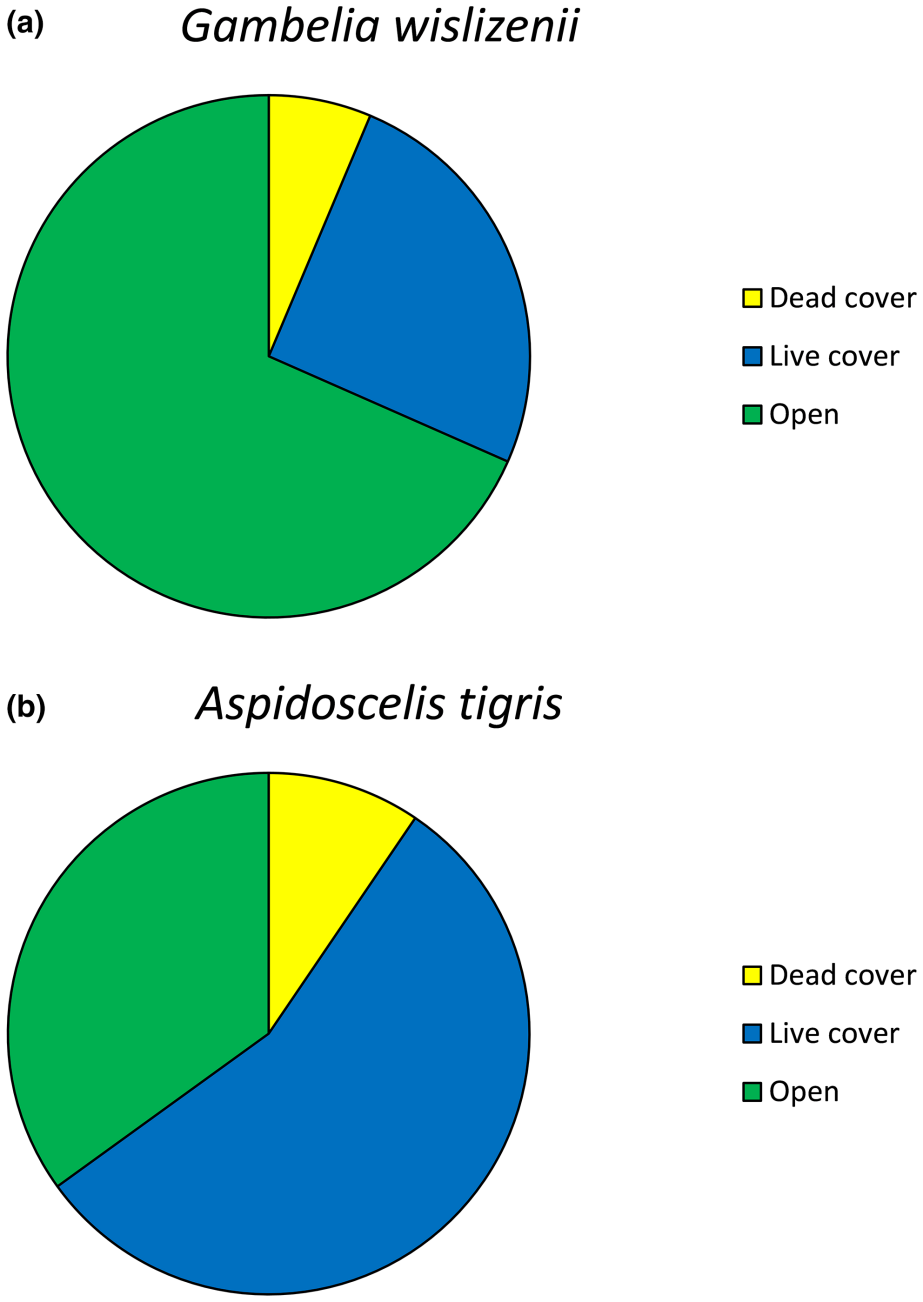
Use of habitat by *Gambelia wislizenii* (visual forager; a) and *Aspidoscelis tigris* (chemosensory forager; b).

**FIGURE 5:**
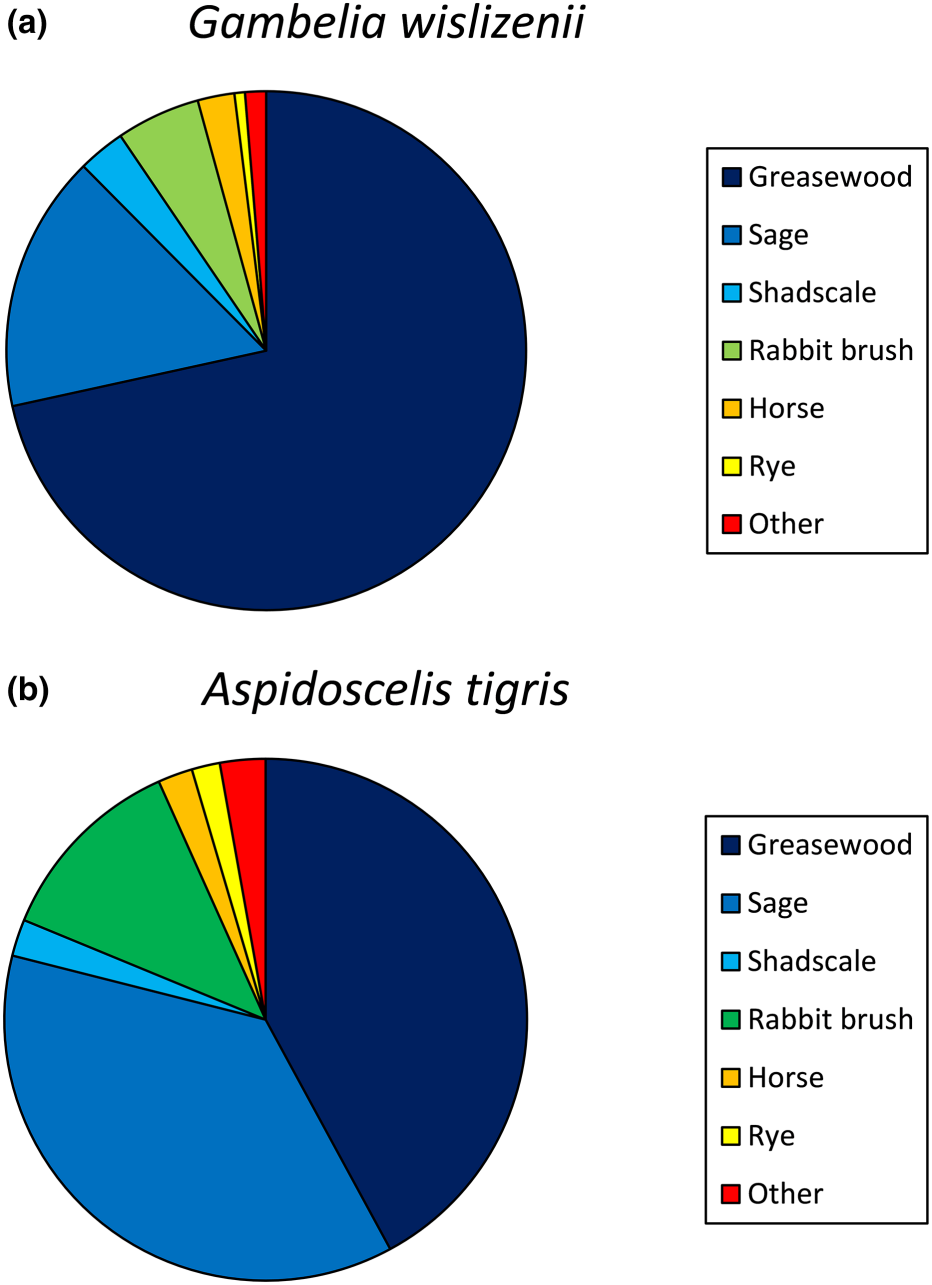
Use of vegetation by *Gambelia wislizenii* (visual forager; a) and *Aspidoscelis tigris* (chemosensory forager; b). Species of plants are in the families Chenopodiaceae (greasewood: *Sarcobatus vermiculatus* and shadscale: *Atriplex confertifolia*), Asteraceae (sage: *Artemisia tridentata*, rabbit brush: *Ericameria* sp., and horse brush: *Tetradymia glabrata*), and Poaceae (ryegrass: *Leymus cincereus*).

The two species varied in time spent under cover, with the visual predator occurring more frequently in the open than the chemosensory predator (*χ* ^2^ = 308.0, df = 2, *p* < .001; Figure 4). Based on differences in the visibility index, the visual predator was more likely than the chemosensory predator to move to locations visible from the previous location (*χ* ^2^ = 392.9, df = 1, *p* < .001). In addition, the two species made use of different species of plants. When under cover, the visual predator primarily used greasewood (*S. vermiculatus*), but the chemosensory predator frequented both sage (*Artemisia tridentata*) and greasewood in roughly equal measure (*χ* ^2^ = 82.1, df = 6, *p* < .001; Figure 5).

## 4 DISCUSSION

Organisms occurring in sympatry must share or partition limited resources to coexist. Although sympatry involves species overlapping in range, species often differ in microhabitat use, activity, or food usage. Sympatric predators can exhibit differences in habitat use and activity (Kozlowski et al., 2006; Parra, 2006; Waite et al., 2012), as well as diet (Hartman & Brandt, 1995). Different foraging modes can lead to sympatric foragers encountering different prey (Huey & Pianka, 1981; O'Brien et al., 1990). Using detailed movement metrics, our study emphasizes behavioral differences between the movements and habitat use of a visual predator that co‐occurs with a chemosensing predator, indicating that movement can play a role in facilitating sympatry and resource partitioning. Although both species exhibited similar net displacement, their vegetation use and patterns of movement varied consistently with the different prey detection strategies. The visually‐oriented animals positioned themselves in open areas where they were able to see prey from a distance, whereas the chemical‐sensing species followed pathways that brought them closer to vegetation, indicative of a reliance on close inspection of chemical cues to acquire prey.

In terms of min‐to‐min spatial advancement, the visually‐oriented *G. wislizenii* showed different movement patterns compared to the chemosensory‐oriented *A. tigris*. Specifically, the visual predator followed our prediction by moving in more direct paths (higher straightness index) and spending more time in the open when compared to the sympatric chemosensory species, a result consistent with the notion that visually‐oriented predators require a clear visual path to stalk prey. We hypothesize that the use of the edges of vegetation and open spaces allows for better visual scans of both plants and open areas, which can increase their prey detection range. The chemosensory predators, in contrast, were seen more frequently under cover which can increase their chances of detecting prey by venturing further into vegetation. The tendency for chemosensory lizards to be found more frequently under cover might also result from predation pressure, as *A. tigris* is vulnerable to both avian predators and larger lizards such as *G. wislizenii* (Steffen & Anderson, 2006).

We recorded our chemosensory predator spending equal time in sage and greasewood bushes, climbing in brush to pursue insect prey, and digging for insect larvae under vegetation. For *A. tigris*, vegetation likely harbors more prey opportunities than open areas but also requires more time to search. The equal occurrence of the chemosensory predator in both shrub types, combined with a consistently less direct trajectory, implies that the whiptails were detecting chemosensory cues while moving from bush to bush, opportunistically searching each bush for prey. For our visual predator, the choice of vegetation might be related to the types of prey likely to be found therein, coupled with the vantage associated with chosen plants. Greasewood, for example, seemed to have a higher branch ceiling than sage (personal observation), possibly proving less of an impediment to visual scanning from a distance. Our study found that the two lizard species use space differently, consistent with efforts to find prey via different foraging methods, in addition to potentially being affected by predator avoidance.

The Alvord Basin study site allowed us to make comparisons of two species and draw novel insights into the interplay between movement and ecology for two sympatric species. While our approach of studying behavioral ecology through an assessment of movement patterns provides a template for examining differences in behavior attributable to species, sex, body size, or season, we acknowledge that our observations might not generalize to other populations or have larger, species‐level implications. Comparing the behavior of the same species in other locations where they occur would promote a more general appraisal of the factors affecting their behavior and sympatry.

## 5 CONCLUSIONS

Path segmentation combined with habitat analyses provides new insight into the ecology of visual and chemosensory prey detection. Individuals of the two species were commonly in proximity to each other, indicating a lack of spatial segregation by species. Yet, they used the same area in very different ways. Landscape features can present a continuum of corridor‐barrier patches acting as functional areas whose use varies by species (Panzacchi et al., 2016). While *G. wislizenii* moved along a straight path to places that readily could be seen from the previous location (high visibility index), *A. tigris* moved more circuitously through vegetation where visibility was lower, demonstrating how landscape features and movement can interact to promote sympatry. Landscape features that facilitate movement for one species can impede movement for the other (Panzacchi et al., 2016). Depending on the overlap in diet, differences in sensory priorities might not, by themselves, prevent competition between species. Their movement, however, could represent a form of resource partitioning that facilitates coexistence.

## AUTHOR CONTRIBUTIONS

**Elizabeth McAlpine‐Bellis:** Conceptualization (supporting); investigation (equal); methodology (equal); visualization (supporting); writing – original draft (supporting); writing – review and editing (lead). **Kaera Utsumi:** Conceptualization (supporting); investigation (equal); methodology (equal); writing – original draft (supporting); writing – review and editing (lead). **Kelly M. Diamond:** Conceptualization (supporting); investigation (equal); methodology (equal); supervision (supporting); writing – original draft (supporting); writing – review and editing (supporting). **Janine Klein:** Conceptualization (supporting); investigation (equal); methodology (equal); writing – original draft (supporting). **Sophia Gilbert‐Smith:** Investigation (equal); methodology (equal). **Grace Elizabeth Garrison:** Investigation (equal); methodology (equal). **Maria A. Eifler:** Conceptualization (lead); data curation (supporting); funding acquisition (supporting); investigation (lead); methodology (lead); project administration (supporting); resources (lead); supervision (lead); visualization (supporting); writing – original draft (supporting); writing – review and editing (lead). **Douglas A. Eifler:** Conceptualization (lead); data curation (lead); formal analysis (lead); funding acquisition (lead); investigation (equal); methodology (equal); project administration (lead); resources (lead); supervision (lead); validation (lead); visualization (lead); writing – original draft (lead); writing – review and editing (supporting).

## FUNDING INFORMATION

Our research was made possible by financial support from Erell Institute and grants from the University of Kansas Honors Program to SGS and the Department of Ecology & Evolutionary Biology Summer Funding Program to GG. Funding agencies played no role in the design of the study, the collection, analysis, and interpretation of data, nor in writing the manuscript.

## CONFLICT OF INTEREST STATEMENT

The authors declare that they have no competing interests.

## Data Availability

Upon publication, all data that support the findings of this study will be deposited in Dryad (Eifler et al., 2023).

## References

[ece310422-bib-0001] Abrahms, B. , Seidel, D. P. , Dougherty, E. , Hazen, E. L. , Bograd, S. J. , Wilson, A. M. , McNutt, J. W. , Costa, D. P. , Blake, S. , Brashares, J. S. , & Getz, W. M. (2017). Suite of simple metrics reveals common movement syndromes across vertebrate taxa. Movement Ecology, 5, 12. 28580149 10.1186/s40462-017-0104-2 PMC5452391

[ece310422-bib-0002] Anderson, R. A. (1993). An analysis of foraging in the lizard, *Cnemidophorus tigris*: Salient features and environmental effects. In J. W. Wright & L. J. Vitt (Eds.), Biology of whiptail lizards (pp. 83–114). Oklahoma Museum of Natural History.

[ece310422-bib-0003] Anderson, R. A. (2007). Food acquisition modes and habitat use in lizards: Questions from an integrative perspective. In S. M. Reilly , L. D. McBrayer , & D. B. Miles (Eds.), Lizard ecology: The evolutionary consequences of foraging mode (pp. 1–10). Cambridge University Press.

[ece310422-bib-0004] Anderson, R. A. , & Karasov, W. H. (1988). Energetics of the lizard *Cnemidophorus tigris*, and life history consequences of food acquisition mode. Ecological Monographs, 58, 79–110.

[ece310422-bib-0005] Attum, O. A. , & Eason, P. K. (2006). Effects of vegetation loss on a sand dune lizard. Journal of Wildlife Management, 70, 27–30.

[ece310422-bib-0006] Baeckens, S. , Van Damme, R. , & Cooper, W. E., Jr. (2017). How phylogeny and foraging ecology drive the level of chemosensory exploration in lizards and snakes. Journal of Evolutionary Biology, 30, 627–640. 28009479 10.1111/jeb.13032

[ece310422-bib-0007] Batschelet, E. (1981). Circular statistics in biology. Academic Press.

[ece310422-bib-0008] Childers, J. L. , & Eifler, D. A. (2015). Intraspecific behavioural variation in the lacertid lizard *Meroles cuneirostris* (Strauch, 1867) (Sauria:Lacertidae). African Journal of Herpetology, 64, 54–66.

[ece310422-bib-0009] Colombo, M. , Indermaur, A. , Meyer, B. S. , & Salzburger, W. (2016). Habitat use and its implications to functional morphology: Niche partitioning and the evolution of locomotory morphology in Lake Tanganyikan cichlids (Perciformes: Cichlidae). Biological Journal of the Linnean Society, 118, 536–550.

[ece310422-bib-0010] Cooper, W. E., Jr. , & Frederick, W. G. (2007). Optimal flight initiation distance. Journal of Theoretical Biology, 244, 59–67. 16949619 10.1016/j.jtbi.2006.07.011

[ece310422-bib-0011] Cooper, W. E., Jr. , & Whiting, M. J. (2000). Ambush and active foraging mode both occur in the scincid genus *Mabuya* . Copeia, 2000, 112–118.

[ece310422-bib-0012] Cooper, W. E., Jr. (1994). Prey chemical discrimination, foraging mode, and phylogeny. In L. J. Vitt & E. R. Pianka (Eds.), Lizard ecology: Historical and experimental perspectives (pp. 95–116). Princeton University Press.

[ece310422-bib-0013] Cooper, W. E., Jr. (1995). Foraging mode, prey chemical discrimination, and phylogeny in lizards. Animal Behaviour, 50, 973–985.

[ece310422-bib-0014] Cooper, W. E., Jr. , Vitt, L. J. , Caldwell, J. P. , & Fox, S. F. (2001). Foraging modes of some American lizards: Relationships among measurement variables and discreteness of modes. Herpetologica, 57, 65–76.

[ece310422-bib-0015] Donihue, C. M. (2016). Aegean wall lizards switch foraging modes, diet and morphology in a human‐built environment. Ecology and Evolution, 6, 7433–7442. 28725410 10.1002/ece3.2501 PMC5513264

[ece310422-bib-0016] Durtsche, R. D. (1992). Feeding time strategies of the fringe‐toed lizard, *Uma inornata*, during breeding and non‐breeding seasons. Oecologia, 89, 85–89. 28313399 10.1007/BF00319019

[ece310422-bib-0017] Edelhoff, H. , Signer, J. , & Balkenhol, N. (2016). Path segmentation for beginners: An overview of current methods for detecting changes in animal movement patterns. Movement Ecology, 4, 1–21. 27595001 10.1186/s40462-016-0086-5 PMC5010771

[ece310422-bib-0018] Eifler, D. A. , & Eifler, M. A. (1999). The influence of prey distribution on the foraging strategy of the lizard *Oligosoma grande* (Reptilia: Scincidae). Behavioral Ecology and Sociobiology, 45, 397–402.

[ece310422-bib-0019] Eifler, D. A. , Eifler, M. A. , & Eifler, E. N. (2007). Habitat use and movement patterns for the lizard, *Pseudocordylus capensis* . African Zoology, 42, 152–157.

[ece310422-bib-0020] Eifler, D. A. , Eifler, M. A. , & Harris, B. (2008). Foraging under the risk of predation in desert grassland whiptail lizards, *Aspidoscelis uniparens* . Journal of Ethology, 26, 219–223.

[ece310422-bib-0021] Eifler, D. A. , Eifler, M. A. , Liu, E. F. , Luyanda, B. , Utsumi, K. , Muradzikwa, T. , Kanyanga, M. K. , & Buchanan, C. A. (2020). Slip slidin’ away: Demographic variation in movement behavior of the dune‐dwelling lizard *Meroles anchietae* . Journal of Arid Environments, 183, 104286.

[ece310422-bib-0022] Eifler, D. A. , McAlpine‐Bellis, E. , Utsumi, K. , Diamond, K. M. , Klein, J. , Gilbert Smith, S. , Garrison, G. E. , Eifler, M. A. (2023). Movement patterns and habitat use for the sympatric species: *Gambelia wislizenii* and *Aspidoscelis tigris* . *Dryad*, Dataset. 10.5061/dryad.rfj6q57gm PMC10413956 37575589

[ece310422-bib-0023] Garrison, G. E. , Zecchini Gebin, J. C. , Penner, J. F. , Jacobson, F. E. , Eifler, M. A. , & Eifler, D. A. (2017). Intraspecific variation in habitat use and movement in long‐nosed leopard lizards, *Gambelia wislizenii* . The Southwestern Naturalist, 62, 187–192.

[ece310422-bib-0025] Greeff, J. , & Whiting, M. J. (2000). Foraging‐mode plasticity in the lizard *Platysaurus broadleyi* . Herpetologica, 56, 402–407.

[ece310422-bib-0026] Grismer, L. L. (2002). Amphibians and reptiles of Baja California. University of California Press.

[ece310422-bib-0027] Hammerson, G. A. (1999). Amphibians and reptiles in Colorado (2nd ed.). University Press of Colorado.

[ece310422-bib-0028] Hartman, K. J. , & Brandt, S. B. (1995). Trophic resource partitioning, diets, and growth of sympatric estuarine predators. Transactions of the American Fisheries Society, 124(4), 520–537.

[ece310422-bib-0029] Higham, T. E. (2007). The integration of locomotion and prey capture in vertebrates: Morphology, behavior, and performance. Integrative and Comparative Biology, 47, 82–95. 21672822 10.1093/icb/icm021

[ece310422-bib-0031] Huey, R. B. , & Pianka, E. R. (1981). Ecological consequences of foraging mode. Ecology, 62, 991–999.

[ece310422-bib-0032] Kays, R. , Crofoot, M. C. , Jetz, W. , & Wikelski, M. (2015). Terrestrial animal tracking as an eye on life and planet. Science, 348, aaa2478. 26068858 10.1126/science.aaa2478

[ece310422-bib-0033] Kozlowski, A. J. , Gese, E. M. , & Arjo, W. M. (2006). Niche overlap and resource partitioning between sympatric kit foxes and coyotes in the Great Basin Desert of western Utah. The American Midland Naturalist., 160(1), 191–208.

[ece310422-bib-0034] Krauel, J. J. , Brown, V. A. , Westbrook, J. K. , & McCracken, G. F. (2018). Predator‐prey interaction reveals local effects of high‐altitude insect migration. Oecologia, 186, 49–58. 29101468 10.1007/s00442-017-3995-0

[ece310422-bib-0035] Leu, S. T. , Jackson, G. , Roddick, J. F. , & Bull, C. M. (2016). Lizard movement tracks: Variation in path re‐use behaviour is consistent with a scent‐marking function. PeerJ, 4, e1844. 27019790 10.7717/peerj.1844 PMC4806635

[ece310422-bib-0036] McElroy, E. J. , McBrayer, L. D. , Williams, S. C. , Anderson, R. A. , & Reilly, S. M. (2011). Sequential analyses of foraging behavior and attack speed in ambush and widely foraging lizards. Adaptive Behavior, 20, 16–31.

[ece310422-bib-0037] McLaughlin, R. L. (1989). Search modes of birds and lizards: Evidence of alternative movement patterns. The American Naturalist, 133, 654–670.

[ece310422-bib-0038] Miles, D. B. , Losos, J. B. , & Irschick, D. J. (2007). Morphology, performance and foraging mode. In S. M. Reilly , L. D. McBrayer , & D. B. Miles (Eds.), Lizard ecology: The evolutionary consequences of foraging mode (pp. 49–93). Cambridge University Press.

[ece310422-bib-0039] Montanucci, R. R. (1967). Further studies on leopard lizards, *Crotaphytus wislizenii* . Herpetologica, 23, 119–126.

[ece310422-bib-0040] Morice, S. , Pincebourde, S. , Darboux, F. , Kaiser, W. , & Casas, J. (2013). Predator‐prey pursuit‐evasion games in structurally complex environments. Integrative and Comparative Biology, 53, 767–779. 23720527 10.1093/icb/ict061

[ece310422-bib-0041] Nathan, R. , Getz, W. M. , Revilla, E. , Holyoak, M. , Kadmon, R. , Saltz, D. , & Smouse, P. E. (2008). A movement ecology paradigm for unifying organismal movement research. Proceedings of the National Academy of Sciences of the United States of America, 105, 19052–19059. 19060196 10.1073/pnas.0800375105 PMC2614714

[ece310422-bib-0042] O'Brien, J. W. , Howard, B. I. , & Barbara, E. I. (1990). Search strategies of foraging animals. American Scientist, 78, 52–160.

[ece310422-bib-0043] Panzacchi, M. , Van Moorter, B. , Strand, O. , Saerens, M. , Kivimäki, I. , St. Clair, C. C. , Herfindal, I. , & Boitani, L. (2016). Predicting the *continuum* between corridors and barriers to animal movements using step selection functions and randomized shortest paths. The Journal of Animal Ecology, 85, 32–42. 25950737 10.1111/1365-2656.12386

[ece310422-bib-0044] Parker, W. S. , & Pianka, E. R. (1976). Ecological observations on the leopard lizard (*Crotaphytus wislizeni*) in different parts of its range. Herpetologica, 32, 95–114.

[ece310422-bib-0045] Parra, G. J. (2006). Resource partitioning in sympatric delphinids: Space use and habitat preferences of Australian snubfin and Indo‐Pacific humpback dolphins. The Journal of Animal Ecology, 75, 862–874. 17009750 10.1111/j.1365-2656.2006.01104.x

[ece310422-bib-0046] Perry, G. (2007). Movement patterns in lizards: Measurement, modality and behavioral correlates. In S. M. Reilly , L. D. McBrayer , & D. B. Miles (Eds.), Lizard ecology: The evolutionary consequences of foraging mode (pp. 13–48). Cambridge University Press.

[ece310422-bib-0047] Pianka, E. R. (1970). Comparative autecology of the lizard *Cnemidophorus tigris* in different parts of its geographic range. Ecology, 51, 703–720.

[ece310422-bib-0048] Pietruszka, R. D. (1986). Search tactics of desert lizards: How polarized are they? Animal Behaviour, 34, 1742–1758.

[ece310422-bib-0049] R Development Core Team . (2017). R: A language and environment for statistical computing. R Foundation for Statistical Computing.

[ece310422-bib-0050] Schick, R. S. , Loarie, S. R. , Colchero, F. , Best, B. D. , Boustany, A. , Conde, D. A. , Halpin, P. N. , Joppa, L. N. , McClellan, C. M. , & Clark, J. S. (2008). Understanding movement data and movement processes: Current and emerging directions. Ecology Letters, 11, 1338–1350. 19046362 10.1111/j.1461-0248.2008.01249.x

[ece310422-bib-0051] Smouse, P. E. , Focardi, S. , Moorcroft, P. R. , Kie, J. G. , Forester, J. D. , & Morales, J. M. (2010). Stochastic modelling of animal movement. Philosophical Transactions of the Royal Society, B: Biological Sciences, 365, 2201–2221. 10.1098/rstb.2010.0078 PMC2894957 20566497

[ece310422-bib-0052] Steffen, J. E. , & Anderson, R. A. (2006). Abundance of the long‐nosed leopard lizard (*Gambelia wislizenii*) is influenced by shrub diversity and cover in southeast Oregon. The American Midland Naturalist, 156, 201–207.

[ece310422-bib-0053] Sunquist, M. E. , & Montgomery, G. G. (1973). Activity patterns and rates of movement of two‐toed and three‐toed sloths (*Choloepus hoffmanni* and *Bradypus infuscatus*). Journal of Mammalogy, 54, 946–954. 4761371

[ece310422-bib-0054] Symes, C. T. , Wilson, J. W. , Woodborne, S. M. , Shaikh, Z. S. , & Scantlebury, M. (2013). Resource partitioning of sympatric small mammals in an African forest‐grassland vegetation mosaic. Austral Ecology, 38(6), 721–729.

[ece310422-bib-0055] Tollestrup, K. (1983). The social behavior of two species of closely related leopard lizards, *Gambelia silus* and *Gambelia wislizenii* . Ethology, 62, 307–320.

[ece310422-bib-0056] Tonini, J. F. R. , Beard, K. H. , Ferreira, R. B. , Jetz, W. , & Pyron, R. A. (2016). Fully‐sampled phylogenies of squamates reveal evolutionary patterns in threat status. Biological Conservation, 204, 23–31.

[ece310422-bib-0057] Utsumi, K. , Kusaka, C. , Pedersen, R. , Staley, C. , Dunlap, L. , Gilbert Smith, S. , Eifler, M. A. , & Eifler, D. A. (2020). Habitat dependent search behavior in the Colorado checkered whiptail (*Aspidoscelis neotesselata*). Western North American Naturalist, 80, 11–18.

[ece310422-bib-0058] Waite, J. N. , Trumble, S. J. , Burkanov, V. N. , & Andrews, R. D. (2012). Resource partitioning by sympatric Steller sea lions and northern fur seals as revealed by biochemical dietary analyses and satellite telemetry. Journal of Experimental Marine Biology and Ecology, 416–417(1), 41–54.

[ece310422-bib-0059] Wasiolka, B. , Blaum, N. , Jeltsch, F. , & Henschel, J. (2009). Behavioral responses of the lizard (*Pedioplanis l. lineoocellata*) to overgrazing. Acta Oecologica, 35, 157–162.

[ece310422-bib-0060] Wasiolka, B. , Jeltsch, F. , Henschel, J. , & Blaum, N. (2009). Space use of the spotted sand lizard (*Pedioplanis l. lineoocellata*) under different degradation states. African Journal of Ecology, 48, 96–104.

[ece310422-bib-0061] Wearmouth, V. J. , McHugh, M. J. , Humphries, N. E. , Naegelen, A. , Ahmed, M. Z. , Southall, E. J. , Reynolds, A. M. , & Sims, D. W. (2014). Scaling laws of ambush predator ‘waiting’ behaviour are tuned to a common ecology. Proceedings of the Royal Society B: Biological Sciences, 281, 20132997. 10.1098/rspb.2013.2997 PMC3973260 24619440

